# Reduction of Motion Artifacts in the Recovery of Undersampled DCE MR Images Using Data Binning and L+S Decomposition

**DOI:** 10.1155/2019/6139785

**Published:** 2019-04-17

**Authors:** Muhammad Bilal, Haris Anis, Najeeb Khan, Ijaz Qureshi, Jawad Shah, Kushsairy A. Kadir

**Affiliations:** ^1^Department of Electrical Engineering Int. Islamic University, Islamabad, Pakistan; ^2^Department of Computer Science, University of Saskatchewan, Canada; ^3^Department of Electrical Engineering, Air University Islamabad, Pakistan; ^4^British Malaysian Institute, Universiti Kuala Lumpur, Kuala Lumpur, Malaysia

## Abstract

**Background:**

Motion is a major source of blurring and ghosting in recovered MR images. It is more challenging in Dynamic Contrast Enhancement (DCE) MRI because motion effects and rapid intensity changes in contrast agent are difficult to distinguish from each other.

**Material and Methods:**

In this study, we have introduced a new technique to reduce the motion artifacts, based on data binning and low rank plus sparse (L+S) reconstruction method for DCE MRI. For Data binning, radial k-space data is acquired continuously using the golden-angle radial sampling pattern and grouped into various motion states or bins. The respiratory signal for binning is extracted directly from radially acquired* k*-space data. A compressed sensing- (CS-) based L+S matrix decomposition model is then used to reconstruct motion sorted DCE MR images. Undersampled free breathing 3D liver and abdominal DCE MR data sets are used to validate the proposed technique.

**Results:**

The performance of the technique is compared with conventional L+S decomposition qualitatively along with the image sharpness and structural similarity index. Recovered images are visually sharper and have better similarity with reference images.

**Conclusion:**

L+S decomposition provides improved MR images with data binning as preprocessing step in free breathing scenario. Data binning resolves the respiratory motion by dividing different respiratory positions in multiple bins. It also differentiates the respiratory motion and contrast agent (CA) variations. MR images recovered for each bin are better as compared to the method without data binning.

## 1. Introduction

DCE MRI provides a measurement of T_1_ changes in tissues over time after the intravenous bolus of CA. It is mostly used as a biomarker in different diseases and oncology [1, 2]. The DCE technique relies on the monitoring of images during uptake and wash out of CA, usually gadolinium. The relationship of pixel intensities and CA concentration can be acquired over time. A pharmacokinetic model based on pixel intensities, CA concentration, and time variation is used to generate kinetic parameter values that may be correlated with the characteristics of tissues [3].

The complete process of DCE-MRI (uptake and washout of CA) takes several minutes and it is difficult for a patient to remain motionless during the scan process. Like other applications of MRI, different types of motions like respiratory motion and heartbeat also affect the DCE-MRI. The* k*-space lines obtained in distinct motion states produce interframe misalignment and the resultant recovered images are blurry and ghosted [4, 5]. In DCE MRI, because of interframe misalignments, it is difficult to differentiate between motion effects and CA changes. Several strategies have been proposed in the literature to avoid the effects of motion. The breath held during MR scan is the simplest and currently used approach to avoid respiratory motion effects [6]. However, suspension of respiration is patient dependent and very difficult in case of paediatrics. References [7, 8] discuss the alternative methods of using navigator and bellows to acquire data at some specific respiratory states, but these techniques are inefficient and prolong the scan timing. Another approach is to acquire the* k*-space data in free breathing and then remove motion artifacts during the image recovery process. Image registration methods for rigid and nonrigid body motion have been applied for cardiac perfusion imaging [9], cardiac cine [10], and abdominal DCE MRI analysis [11]. These methods exploit the high acceleration rate capability of compressed sensing [12–14] for the recovery of dynamic MRI. Most of the motion estimation and correction methods, based on data binning in different respiratory states, are ECG gated [10, 15]. Currently research is focused on real time MRI, in which respiratory signal is extracted from observed* k*-space data for data binning [16–18]. The radial sampling scheme is more suitable for real time MRI because of its robustness for motion, and repeated sampling of the* k*-space centre provides information for the extraction of the respiratory signal directly from acquired data [18, 19].

L+S decomposition (also known as robust principle component analysis (RPCA)) is an actively used technique for CS-based dynamic MRI. It represents the dynamic MR images as a superposition of a slowly varying background component and a rapidly changing foreground dynamic component [20]. Gao et al. [21, 22] have discussed a combined approach of CS and L+S decomposition for the recovery of retrospectively subsampled cardiac cine data and a series of diffusion-weighted images. Otazo et al. applied L+S decomposition to recover accelerated dynamic cardiac perfusion and free breathing DCE MR images [20]. They recovered the final images by combining CS and L+S decomposition without any preprocessing step for respiratory motion.

In DCE MRI, the contrast agent produces strong intensity variations over time. This intensity change is another challenge along with respiratory motion in free breathing DCE MRI. To generate a good quality MR image, intensity variation and respiratory motion must be distinguished from each other before using registration-based methods or CS-based L+S decomposition method.

In this study, the data binning process is reported for the first time as a preprocessing method, to distinguish intensity changes from respiratory motion, for CS-based L+S decomposition method. As compared to the CS-based conventional L+S (CL+S) decomposition method, the preprocessed L+S method, named here the hybrid L+S (HL+S) method, provides improved results in a free breathing environment for 3D abdominal and liver DCE MRI. In the preprocessing step, motion detection and extraction of a breathing signal by exploiting the self-navigation property of golden-angle radial sampling are performed as adopted in [23, 24]. The extracted respiratory signal is segmented into different contrast phases. The subrespiratory signal for each contrast phase is then sorted in multiple respiratory states (bins). All bins have an equal number of radial spokes with very little respiratory motion. A CS-based L+S decomposition algorithm is used for each motion state (bin) to recover images, free from motion and undersampling artifacts. The HL+S method outperforms the CL+S decomposition technique qualitatively as well as in terms of structural similarity index and image sharpness.

## 2. Related Material

### 2.1. Automatic Motion Detection and Data Binning

The basic strategy is to acquire DCE-MR images with respiratory motion and then reconstruct the images free from motion effects. To recover motion free images, acquired* k*-space samples must be grouped in such a way that each group (motion state) has very little respiratory motion. For grouping or binning of the data, two principle requirements are (a) reliable respiratory motion signal and (b) uniform coverage of -space after data b* k* inning. Consecutive spokes of the golden-angle radial sampling with an angle of approximately 111.25° are used for data acquisition [25]. This sampling scheme samples the* k*-space centre repeatedly which enables the extraction of respiratory motion state signals [23, 24] for data binning. It also provides consistent* k*-space coverage in all respiratory states with adequate randomness in sampling pattern for the application of compressed sensing. The 3D DCE-MRI* k*-space data can be represented in matrix form as follows:

(1) $$\begin{array}{c} m_{u}=F_{nu}Cx \end{array}$$

where **x** is the 3D DCE image series to be recovered with (*x* − *y* − *z* − *N*_*c*_) dimensions, **F**_*nu*_ is the nonlinear fast Fourier transform operator (NUFFT), C=C1⋮CNc represents the coil sensitivity maps for *N*_*c*_ number of coils in *x* − *y* space, and **m**_*u*_ is the unsorted multicoil radially sampled* k*-space data with (*N*_*r*_ − *N*_*s*_ − *z* − *N*_*c*_) dimensions. *z* represents the linear slice dimension, *N*_*r*_ is the number samples along a spoke, and *N*_*s*_ is the number of spokes.

A robust approach, for the detection of motion from* k-*space data, is to use projections along the slice dimension for 3D stack of star imaging [26]. In this approach, spokes for all slices along *z* direction are acquired and then 1D Fourier transform is computed to obtain the projection profiles for central points (*x*, *y* = 0) and for all acquisition angles. Once the projection profile obtained for all coils, they are linked to form the following (*N*_*c*_ × *N*_*r*_) − *by* − *N*_*s*_ matrix:

(2) A=U1⋮UNc,  with  Uα=uαa1,b1…uαa1,bNs⋮⋱⋮uαaNr,b1…uαaNr,bNs for  α=1,2…Nc

**Figure 1:**
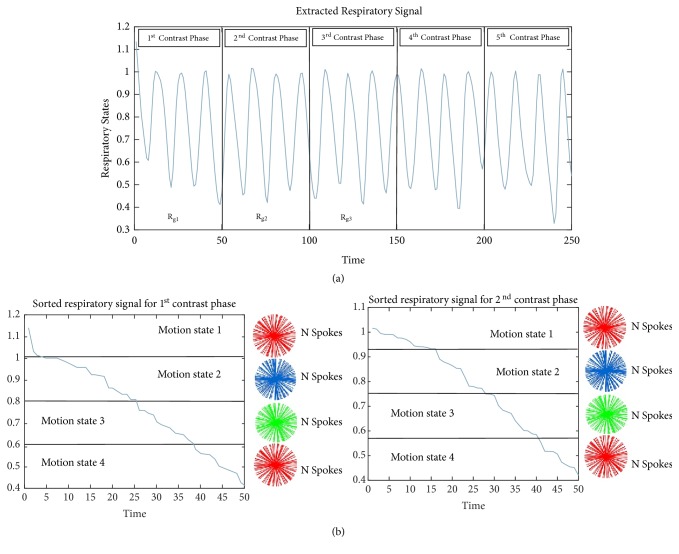
For liver DCE-MRI, extraction and binning of respiratory signal (a) Extracted respiratory signal from k-space data. The signal is divided into different contrast phases. (b) Binning procedure for the sorted respiratory motion signal carried out in every contrast enhancement phase separately. Distinct radially sampled patterns (shown in different colours) are used for different respiratory states and the same number of spokes is used for each respiratory state.

As discussed in [23], PCA is used to estimate the motion signal from concatenated data matrix *A* of all coils. PCA is accomplished by computing the right singular vectors of A, or equivalently the Eigen-vectors of the covariance matrix *Cov* = *A*^*T*^*A*. Principle component with highest peak in the range of respiratory signal frequency 0.1Hz to 0.5Hz is selected to represent the breathing signal. Based on the estimated respiratory signal, the radially acquired data is first divided into consecutive contrast enhancement phases and every phase is then further divided into multiple motion states having the same number of spokes. This binning process provides the data **d** with dimension (*N*_*r*_ − *N*_*s*_ − *N*_*cont*_ − *N*_*R*_), where *N*_*cont*_ shows the number of contrast phases and *N*_*R*_ represents number of respiratory states. The idea is shown in Figure 1. Contrast phases mean the different frames representing intensity variations in CA with respect to time. R_g1_, R_g2_ etc. are the subrespiratory signals which are sorted for data binning as shown in Figure 1(b).

**Figure 2:**
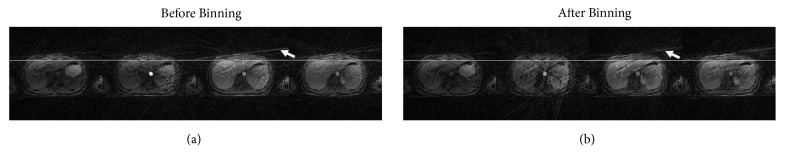
Contrast enhancement phases before and after binning. (a) Motion is present along with streaking artifacts. The solid line clearly shows the misalignment of different contrast phases. (b) Respiratory motion is resolved after binning and phases are aligned with each other. It can be observed along the solid line. White arrows show streaking artifacts.

Binning of radially sampled k-space date in different respiratory states clearly reduces the motion effects (can be observed by solid line in Figure 2) but on the other hand introduces streaking artifacts (shown by white arrows). L+S decomposition in conjunction with compressed sensing is used to remove these undersampling artifacts.

### 2.2. L+S Matrix Decomposition

The L+S matrix decomposition for DCE MRI decomposes it into a low rank component, containing smooth and slow variations and a sparse component comprising fast and local intensity changes. A necessary condition for this decomposition is incoherence between **L** and **S** components. It means that **L** component should not be sparse and sparse component should not have a low rank [27, 28].

For the implementation of L+S decomposition method, the sequence of DCE MR Images is placed in a matrix form such that each column represents one temporal frame. This matrix is called Casorati matrix. The L+S decomposition is performed by solving the following convex optimization problem:

(3) min L∗+λS1s.t. M=L+S

where **S** represents sparse matrix, **L** is the low rank matrix, and **M** is the Casorati matrix. ‖**L**‖_*∗*_ is the nuclear norm (the sum of singular values of **L**), ‖**S**‖_1_ is the* l*_1_-norm (the sum of absolute values of components of **S**), and *λ* is a balancing parameter that defines the share of* l*_1_-norm relative to the nuclear norm.

**Figure 3:**
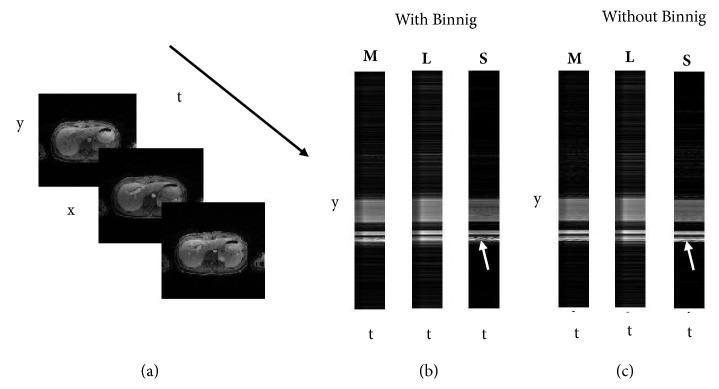
L+S decomposition for DCE MRI with and without binning (a) series of images along time. Decomposed components in y-t space (b) for proposed method (c) without binning. The S component is sparser in (b) as compared to the S component in (c).

Figure 3 shows the L+S decomposition of DCE MRI data set after binning, where **L** captures the smooth and slow varying correlated background between frames and **S** captures the contrast enhancement changes. Two features can be observed in Figures 3(b) and 3(c). The first one is the S component which is sparser than **M** component for both data sets. The separation of smooth changes from contrast enhancements provides gain in sparsity and in principle permit higher acceleration rates [20]. The second one is the **S** component of the proposed method (Figure 3(b)) which is sparser than the **S** component for the data set without binning (Figure 3(c)). This increased sparsity is achieved by resolving the motion through the binning process. As a result, higher acceleration factors can be achieved with the proposed method.

### 2.3. Under Sampled MR Images Reconstruction

The modified version of (4) for radially undersampled liver DCE MR data set **d** can be written as

(4) min L∗+λψS1s.t. d=EL+S

and the unconstrained version of (4) can be given as

(5) min 12EL+S−d22+λLL∗+λSψS1

where **ψ** is the sparsity inducing transform applied to **S** and **d** is the radially undersampled data obtained after binning process as discussed above. **E** is the multiple receiver coil encoding operator, which includes coil sensitivities **C** and undersampled NUFFT **F**_*nu*_ [29]. These factors are multiplied to get **E**, as described in the iterative Sensitivity Encoding (SENSE) algorithm [30]. The multicoil reconstruction approach gives better performance due to the enforcement of joint multicoil low rank and sparsity [9]. *λ*_*L*_ and *λ*_*S*_ are trade-off parameters and provide a balance between data consistency term and the other two terms (nuclear and* l*_1_ norm). The optimization problem in (5) is solved by combining singular value thresholding, a method used for matrix completion [31], and iterative soft thresholding used for sparse signal recovery [32]. The shrinkage or soft thresholding is defined as

(6) $$\begin{array}{c} T_{\beta }\left(\;v\;\right)=\frac{v}{\left|v\right|}\max\left(\;\left|\;v\;\right|-\beta ,0\;\right) \end{array}$$

For matrices, the soft thresholding is applied to every entry. Next we define the singular value thresholding (SVT) by

(7) $$\begin{array}{c} {SVT}_{\beta }\left(\;M\;\right)=UT_{\beta }\left(\;\Sigma \;\right)V^{H} \end{array}$$

where *M* = *U*Σ*V*^*H*^ is singular value decomposition of Casorati matrix *M*.  Algorithm 1 shows the proposed HL+S algorithm for the recovery of DCE MR Images.

## 3. Methods

The performance of the proposed methodology was tested for undersampled 3D liver and abdominal DCE-MRI with respiratory motion for a number of subjects. The human imaging was performed after the approval from the institutional review board (IRB). Written informed consent was obtained from all subjects before imaging studies.

Golden-angle radial sampling, with 111.25° angular increment between consecutive spokes [25], was used for data acquisition. MATLAB (Mathswork, Natik, MA) was used for image reconstruction. The multicoil encoding operator **E** was implemented using NUFFT [29] because of radial sampling. Adaptive coil combination method discussed in [33] was used to generate coil sensitivity maps. The balancing parameters *λ*_*s*_ and *λ*_*L*_ were chosen empirically by comparing reconstructed images for different values. Results for both data sets were generated using the modified version of nonlinear conjugate algorithm [14]. The performance of the proposed method was assessed qualitatively as well as by sharpness index (SI), a measuring parameter based on image sharpness, and is given as [34]

(8) $$\begin{array}{c} SI\left(\;I\;\right)=-\log\phi \left(\;\frac{m-TV\left(I\right)}{v}\;\right) \end{array}$$

where *m* = *𝔼*[(*TV*(*I*))] is the expected value of the total variation of the recovered image *I*, *v* = *𝕍*[(*TV*(*I*))] is the corresponding variance, and *ϕ* is the normal distribution tail as given in [34]. Structural similarity index (SSIM) is used to measure the similarity between recovered images and reference images [35].

### 3.1. Free Breathing 3D Abdominal DCE-MRI

3D abdominal imaging was carried out on a healthy volunteer on an entire-body 3.0T scanner (Siemens AG, Erlangen, Germany) having standard 12-element body matrix coil. The 3D stack-of-stars (radial sampling for *x*, *y* = 0 and Cartesian sampling for *z*) pulse sequence with golden-angle acquisition method was used to acquire data in transversal orientation. Intravenous injection of 10 mL of gadopentetate dimeglumine (Gd-DTPA) (Magnevist; Bayer Healthcare, Leverkusen) was started at a time with the beginning of data acquisition. The process was completed by injecting 20 mL saline for flushing purpose. Injection rate was 2 mL/s for both contrast agent and saline. For a single scan, the imaging parameters for the volunteer were repletion time TR/echo time TE = 3.52/1.41 ms, FOV = 360x360x240 mm^3^, number of readout points in each spoke = 256, spatial resolution = 1.4x1.4x3 mm^3^, and number of partitions = 80, with 60% slice resolution reduction and 6/8 partial Fourier applied to the slice dimension. A total of 600 spokes were continuously acquired in each partition, for a total scan time of 95 s.

### 3.2. Free Breathing 3D Liver DCE MRI

3D liver DCE-MRI was performed at four volunteers on an entire-body 3.0T scanner (Siemens AG, Erlangen, Germany) fitted with the standard 12-element body matrix coil. The 3D stack-of-stars (radial sampling for *x*, *y* = 0 and Cartesian sampling for *z*) pulse sequence with golden-angle acquisition method was used to acquire data in transversal orientation. For each scan, a weight-based half-dose injection (0.1 mmol per kilogram of body weight) of Magnevist (Bayer Healthcare, Berlin, Germany) was performed 20s after the start of data acquisition, at a rate of 2 mL/s. The imaging parameters were repetition time (TR)/echo time (TE) = 3.6/1.6 ms, matrix size = 256x256x48, FOV= 350x350x240 mm^3^, acquired voxel size = 1.37x1.37x5.0 mm^3^, and flip angle = 12°. Eighty percent partial Fourier was applied to the slice dimension and a total of 1222 spokes were obtained for every partition, resulting in a total scan time of 190 s.

## 4. Results

### 4.1. Free Breathing 3D Abdominal DCE-MRI

**Figure 4:**
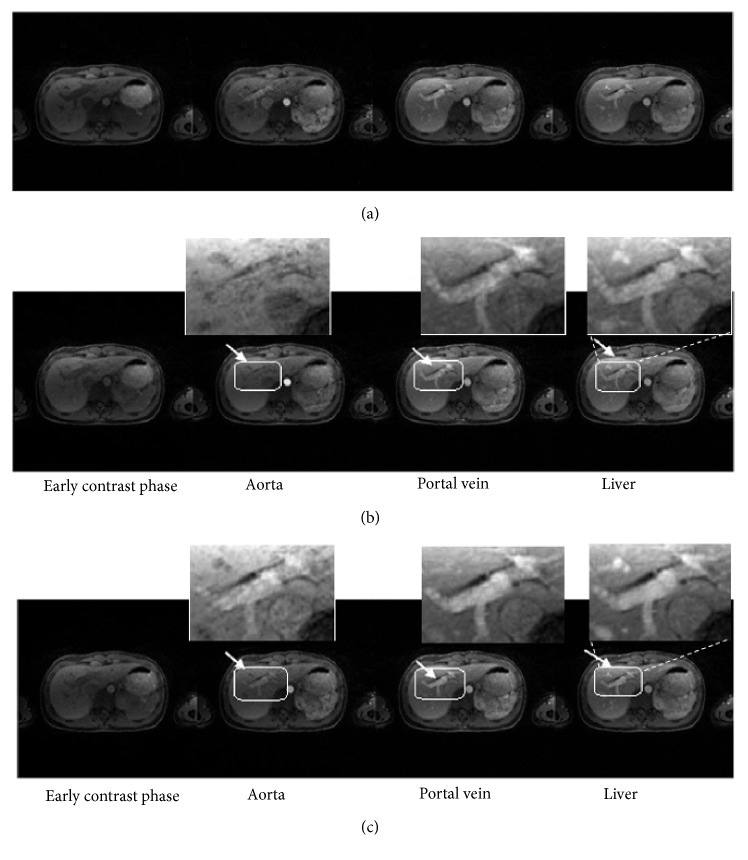
Qualitative comparison: reference images (a) CL+S images (before binning) (b) and HL+S images (after binning) (c) for abdominal DCE MRI. Improved vessels illustration and removal of blurring effects from contrast phases can be observed in the magnified view in (c).

Figure 4 provides a comparison between HL+S method and CL+S.  Figure 4(a) is the reference image representing four contrast phases for a volunteer data set. This single slice taken from 11 slices of complete data set corresponds to early contrast phase, aorta, portal vein, and liver. Structural similarity is measured with respect to this reference image.

Proposed technique presents improved reconstruction performance in all four phases as compared to CL+S, as pointed out by a better representation of small structures that appear blurry in Figure 4(b) (white arrows). Vessels and tissue contrast improvement can also be observed in Figure 4(c). The magnified view of area bounded by white boxes provides a better comparison between HL+S and CL+S methods.

**Figure 5:**
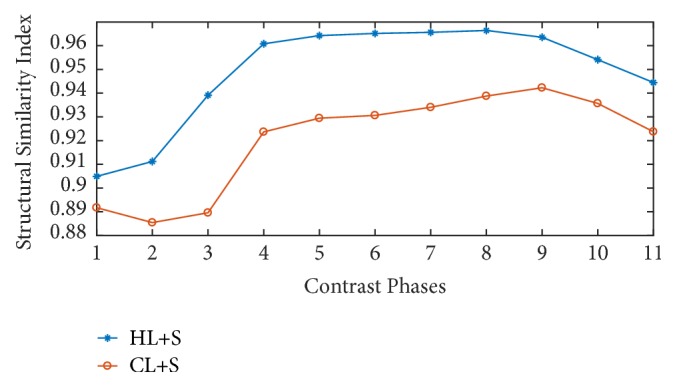
Structural similarity based performance comparison for different contrast phases.

Figure 5 presents the similarity of images, recovered before and after binning, with respect to the reference image given in Figure 4(a). Higher similarity indices can be observed for the proposed method. This higher value of SSIM is achieved due to the preprocessing step of binning which reduces the motion effects in recovered images.

**Figure 6:**
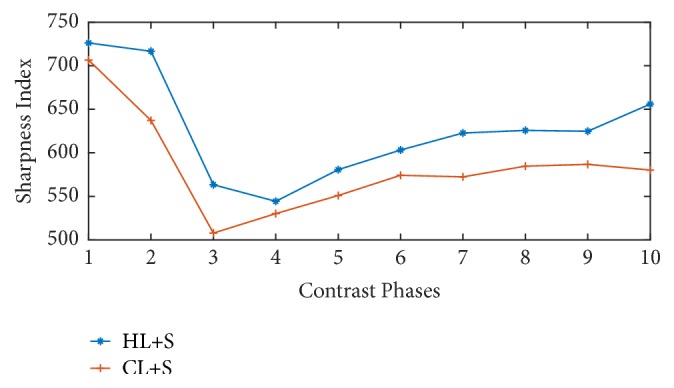
Sharpness indices comparison of contrast phases recovered with and without binning. Higher sharpness index can be observed for the proposed method.

The sharpness indices comparison between conventional L+S method and proposed technique is given in Figure 6. It is clear from the plot that proposed technique (HL+S) outperforms the CL+S decomposition method.

### 4.2. Free Breathing Liver DCE MRI

**Figure 7:**
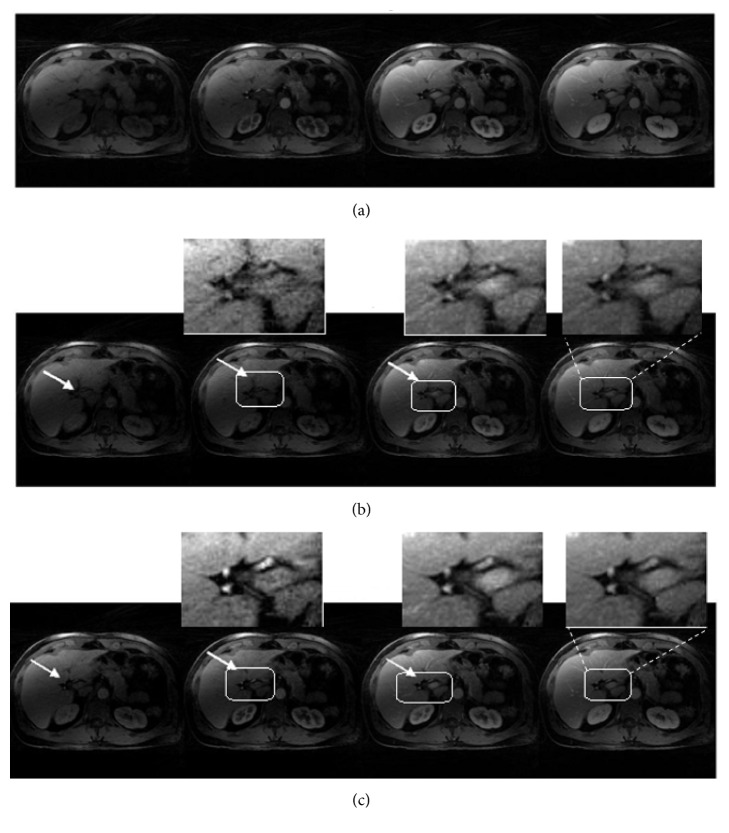
Qualitative comparison: reference images (a) CL+S images (before binning) (b) and HL+S images (after binning) (c) for liver DCE MRI. Without binning recovery suffered from respiratory motion blurring effects. In contrast, the proposed method enabled improved reconstruction of all phases, better capture of the arterial phases, and higher vessel clarity and sharpness. These effects can be observed in magnified views.

Figures 7 and 10 show different contrast enhancement phases recovered by the proposed method and CL+S decomposition method along with reference images in (a). The preprocessing step improved the illustration of vessels and vessel-tissue contrast in the recovered images with the HL+S method as compared to the CL+S method. Comparison points are shown by White arrows and boxes in (b) and (c).

**Figure 8:**
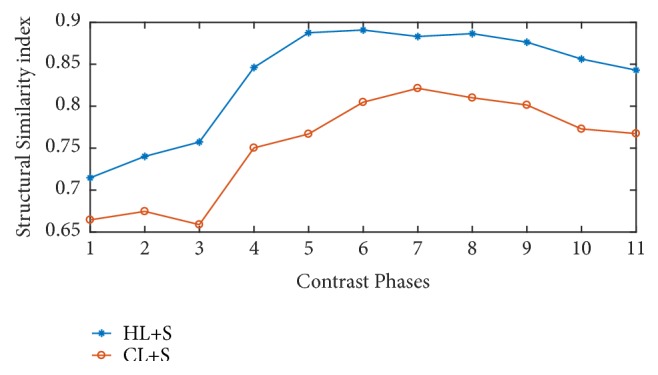
Structural similarity based performance comparison for different contrast phases.

**Figure 9:**
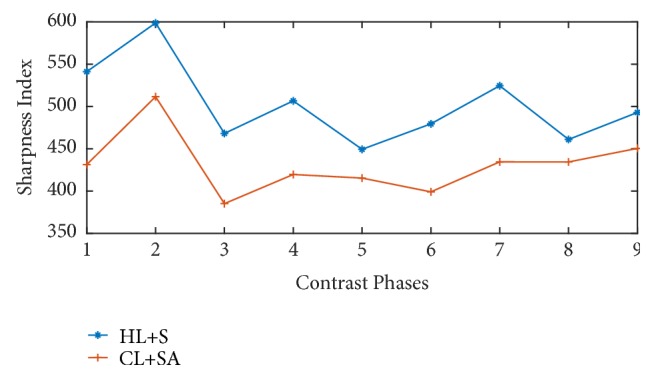
Sharpness indices comparison of contrast phases recovered with and without binning. Higher sharpness index can be observed for the proposed method.

**Figure 10:**
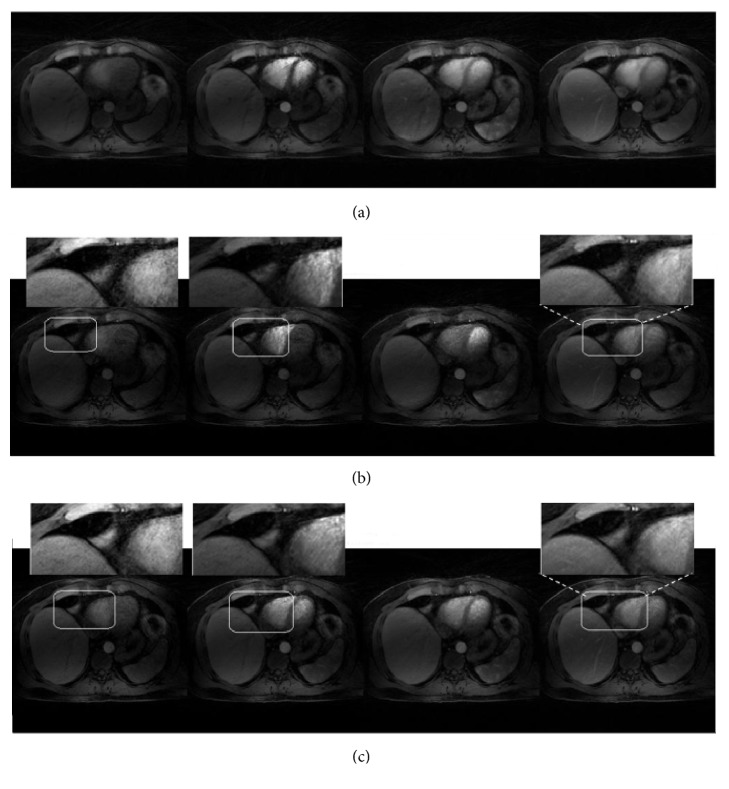
Qualitative comparison: reference images (a) CL+S images (before binning) (b) and HL+S images (after binning) (c) for liver DCE MRI. The magnified view of different phases with binning is visually improved compared to the results obtained without binning.

Figure 8 provides structural similarity indices comparison and Figure 9 presents sharpness indices comparison between HL+S and CL+S method. For parameters, sharpness, and SSIM, the proposed HL+S method performs better as compared to CL+S method.

## 5. Discussion

**Figure 11:**
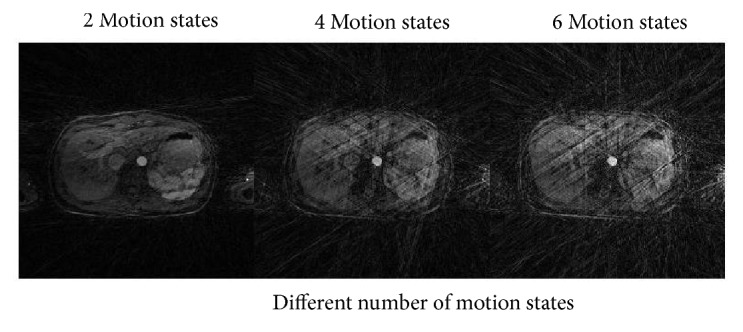
Comparison of a few different respiratory states. 2 motion states have less streaking artifacts as compared to 4 and 6 respiratory motion states.

Data binning provides a new method to deal respiratory motion in free breathing DCE MRI. Sorting the data in similar motion state reduces most of the free breathing artifacts in recovered images. No interpolation errors occurred in the proposed technique and provide a great advantage over the CS-based registration techniques [10, 36], which uses an image registration process in multiple respiratory states to correct motion. The number of motion states to resolve the respiratory motion is selected empirically. A trade-off must be adopted between visualization of breathing motion through binning and undersampling artifacts. By increasing motion states, the number of radial spokes for each state will be reduced and hence increasing the undersampling artifacts. On the other hand, when we choose less number of motion states the undersampling artifacts are reduced because more spokes are available for each state. But the motion is not resolved effectively in this case.  Figure 11 shows the effects of the number of motion states on images recovered through conventional NUFFT without CS-based L+S decomposition method.  Figure 11 shows that respiratory motion is resolved better for 4 and 6 motion states as compared to 2 motion states, but the undersampling artifacts for 2 motion states are very small in comparison with the other two states.

The golden-angle radial undersampling scheme for data binning increases the computational cost mainly due to the computation of NUFFT in forward and backward direction during each iteration. This problem can be resolved by parallel computational techniques. The reconstruction algorithm uses balancing parameters *λ*_*L*_ and *λ*_*S*_. They are selected empirically from a range of values that give the best image quality. The selection process for these parameters is lengthy for dynamic imaging method, but once found, similar parameter values can be used for data sets having same dynamic information. Automatic selection of regularization parameter as discussed in [37] is also applicable to L+S decomposition method. *λ*_*L*_ and *λ*_*S*_ parameters balance the contribution of the **L** and **S** components. Since we are interested in overall reconstruction not in **L** or **S** separately, the technique is less sensitive to the selection of balancing parameters.

## 6. Conclusion

In this research article, data binning is used as a preprocessing step to reduce motion effects with conventional L+S decomposition. For binning, respiratory motion signal is extracted directly from radially sampled data. The preprocessing provides **S** component with greater sparsity which results in higher reconstruction performance. The separation of background and dynamic information provided by L+S decomposition is improved by proposed method without the need for motion correction. The recovered images have better sharpness, clarity, and similarity with reference images as compared to conventional L+S decomposition method.

## Figures and Tables

**Algorithm 1:**
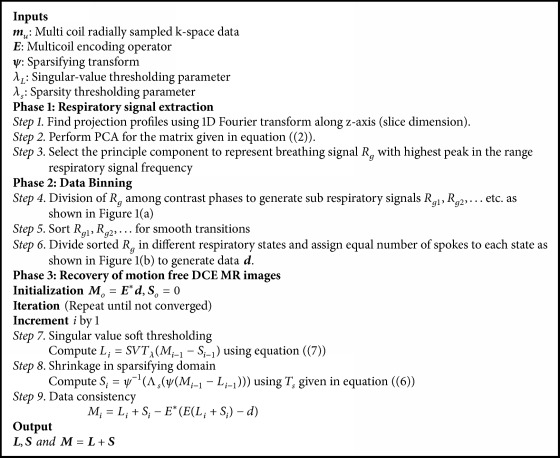
Proposed hybrid L+S (HL+S) reconstruction algorithm for DCE MRI.

## Data Availability

The data used to support the findings of this study are included within the article.
